# Toll-Interacting Protein Suppresses HIV-1 Long-Terminal-Repeat-Driven Gene Expression and Silences the Post-Integrational Transcription of Viral Proviral DNA

**DOI:** 10.1371/journal.pone.0125563

**Published:** 2015-04-27

**Authors:** Fu-Chun Yang, Wen-Dong Kuang, Chuan Li, Wei-Wei Sun, Di Qu, Jian-Hua Wang

**Affiliations:** Key Laboratory of Molecular Virology and Immunology, Institute Pasteur of Shanghai, Chinese Academy of Sciences, Shanghai, China; George Mason University, UNITED STATES

## Abstract

Toll-interacting protein (Tollip) is a host adaptor protein for negatively regulating Toll-like receptor 2-, 4-, and IL-1R (interleukin-1 receptor)-mediated signaling. We found that Tollip expression could be induced in MDDCs (monocyte-derived dendritic cells) by HIV-1 particles and recombinant gp120 glycoprotein. Hence, we investigated the role of Tollip in modulating HIV-1 infection. We found that Tollip expression suppressed NF-κB-dependent HIV-1 long terminal repeat (LTR)-driven transcription and thus inhibited HIV-1 infection. Our protein truncation experiments proved that the intact C-terminus of Tollip was required for inhibition of both NF-κB activity and HIV-1 LTR-driven gene expression. Intriguingly, Tollip silenced the post-integrational transcription of HIV-1 proviral DNA, indicating the potential role of Tollip in maintaining viral persistence. Our results reveal the novel role of host factor Tollip in modulating HIV-1 infection, and may suggest the hijacking of Tollip as the negative regulator of the TLR pathway and even the downstream signaling, by HIV-1 for maintaining persistent infection. Further elucidation of the mechanisms by which HIV-1 induces Tollip expression and identification of the role of Tollip in modulating HIV-1 latency will facilitate the understanding of host regulation in viral replication and benefit the exploration of novel strategies for combating HIV-1 infection.

## Introduction

The innate immune system plays a significant role in fighting against invading pathogens [1–4]. The pattern recognition receptors (PRRs) expressed in immune cells, such as membrane-bound-toll-like receptors and cytoplasmic NOD-like receptors, can identify pathogen-associated molecular patterns for priming host immunity and inducing interferon-stimulated genes to combat pathogens [5–8].

Toll like receptor (TLR) signaling has been shown to inhibit HIV-1 and other retroviral infections. The triggering of TLR2-5 and -8 with agonists blocks HIV-1 and simian immunodeficiency virus replication in macrophages [9–11]. However, TLR signaling can also be manipulated by HIV-1 to avoid recognition for immune evasion. The trans-membrane domain (TMD) of the HIV-1 envelope directly interacts with the TLR2 TMD within the membrane milieu to attenuate agonist-induced cytokine secretion[12]. Single-nucleotide polymorphisms in TLR genes TLR2-4 and TLR6-9 have been previously shown to be associated with HIV-1 acquisition and disease progression in various populations[13–18]. Understanding the modulation of HIV-1 with TLR signaling will benefit the understanding of host restriction and viral pathogenesis.

Toll-interacting protein (Tollip) is an inhibitory adaptor protein within interleukin-1 receptor (IL-1R) and TLR signaling [19–21]. Tollip negatively regulates NF-κB and JNK signaling by inhibiting IL-1R-associated kinase (IRAK) phosphorylation in a MyD88-dependent manner [19–21]. Tollip prevents IFN-α- and TNF-α-induced caspase-8-dependent apoptosis by downregulating TLR2 expression [22]. Moreover, Tollip plays an important role in protein trafficking by interacting with Tom1, clathrin, and ubiquitin[23–26]. Recently, it was reported that Tollip is involved in the polyglutamine protein in Huntington’s disease aggregation and clearance through an ubiquitin-Agt8dependent autophagy[27,28].

In this study, we found that Tollip could be induced by HIV-1 particles and recombinant gp120 glycoproteins for expression in monocyte-derived dendritic cells (MDDCs). We thus investigated the role of Tollip in modulating HIV-1 infection. We found that Tollip suppressed NF-κB-dependent HIV-1 LTR-driven transcription, and that the silence of Tollip in the post-integration transcription of HIV-1 proviral DNA may indicate the potential role of Tollip in maintaining viral persistence.

## Materials and Methods

### Ethics statement

The Medical Ethics Review Committee of Institute Pasteur of Shanghai, Chinese Academy of Sciences has approved the usage of human cells.

### Plasmids

The human full-length *Tollip* gene encoding 274 amino acids was cloned and inserted into the plasmids of pCMV-Tag3B and pCDH-CMV-flag, and the later lentivirus vector was used to generate stable Tollip-expressing clones in THP-1 cells. The C-terminal truncation mutants of aa1-228 and aa1-178 were constructed from the pCMV-Tag3B/Tollip plasmid with specific primers and molecular technology. The full length sequence of HIV-1/NL4-3-LTR was cloned into the pGL3-luc reporter plasmid. The 3κB-luc NF-κB reporter plasmid was kindly donated by Dr. Chen Wang (Shanghai Institute of Biochemistry and Cell Biology, CAS, Shanghai, China) and was described previously [29]. The pCMV-Tat was kindly donated by Dr. Li Wu (Ohio State University, USA)[30]. Lipofectamine 2000 (Life Technologies) was used for transfection according to the manufacturer’s protocol.

### Cell lines and primary cells

HEK293T cells and TZM-bl cells were cultured in Dulbecco modified Eagle medium (DMEM) supplemented with 10% fetal bovine serum (FBS) and penicillin-streptomycin. The TZM-bl cell line was kindly donated by Dr. Paul Zhou (Institute Pasteur of Shanghai, Chinese Academy of Sciences, China)[31]. THP-1/Tollip stable cells were constructed with pCDH-Tollip-flag and cultured in the presence of puromycin (2 μg/mL).

Peripheral blood mononuclear cells (PBMCs) were isolated from the buffy coat of healthy donors, purchased from the Blood Center of Shanghai (Shanghai, China). CD14^+^ monocytes and CD4^+^ T cells were purified from PBMCs using anti-CD14- and anti-CD4-specific antibody-coated magnetic beads (Miltenyi Biotec), respectively, as described previously[32,33]. Monocyte-derived dendritic cells (MDDCs) were differentiated from the CD14^+^ monocytes in culture with interleukin-4 (IL-4) and the granulocyte macrophage colony-stimulating factor (GM-CSF) (RD Systems) for 5 days[34]. Primary CD4^+^ T cells were cultured in the presence of IL-2 (RD Systems). Some cells were stimulated with phytohemagglutinin-P (PHA-P) (Sigma-Aldrich) for 72 h.

### Virus stocks and recombinant gp120 glycoproteins

Single-cycle infectious HIV-1 (HIV-luc/JRFL and HIV-luc/VSV-G) virus stocks were generated by calcium phosphate-mediated co-transfection of HEK293T cells with the HIV-1 plasmid pLAI-Δenv-Luc and a packing plasmid, either pJRFL or pVSV-G, as previously described [33,34]. HIV-1 virus like particles (VLPs) were produced by co-transfection of HEK293T cells with a Gag expression plasmid pHIV-Gag-GFP and a packing plasmid pJRFL or pHXB2. These plasmids were kindly donated by Dr. Li Wu (The Ohio State University, USA). All virus stocks were normalized with p24^gag^ capture ELISA and stored at -80°C. Recombinant gp120 glycoproteins derived from either HIV-1/JRFL or HIV-1/HXB2 (eEnzyme) were used in some of the experiments.

### Reverse transcription and real-time PCR

Total cellular RNA was isolated by using the TRIzol reagent (Life Technologies). The RNA was reverse transcribed into cDNA using the PrimeScript RT Reagent Kit with gDNA Eraser (Takara). Total cellular DNA samples were extracted with the QIAamp DNA Blood Mini Kit (Qiagen). Real-time PCR was performed using the Thunderbird SYBR qPCR Mix (Toyobo) with pre-denaturation at 95°C for 1 min, amplification with 40 cycles of denaturation (95°C, 15 s), and annealing (60°C, 45 s). The data were analyzed by green-based SYBR, semi-quantified and normalized with β-Actin. Real-time PCR was performed on the ABI 7900HT Real-Time PCR system. The primers used are listed below: TNFα-F: 5'- CCTGCCCCAATCCCTTTATT -3', TNFα-R: 5'- CCCTAAGCCCCCAATTCTCT -3'; IL-6-F: 5'- AGACAGCCACTCACCTCTTCAG -3', IL-6-R: 5'- TTCTGCCAGTGCCTCTTTGCTG -3'; IL-10-F: 5'- TCTCCGAGATGCCTTCAGCAGA -3', IL-10-R: 5'- TCAGACAAGGCTTGGCAACCCA -3'; β-Actin-F: 5'- GGGAAATCGTGCGTGACAT -3', β-Actin-R: 5'- GTCAGGCAGCTCGTAGCTCTT -3'. Gag-F: 5'- GTGTGGAAAATCTCTAGCAGTGG -3', Gag-R: 5'- CGCTCTCGCACCCATCTC -3'.

### Transfection of siRNA

MDDCs were transfected with either Tollip-specific siRNA (siTollip) or the off-target control using lipofectamine 2000 (Life Technologies). 72 h post-transfection, cells were harvested for western blotting to detect Tollip expression or viral infection. The siTollip and off-target control were produced by GenePharma Inc. (Shanghai, China). The following oligos were used: siTollip-1: GCAAAGUGGCCAAGAAUU, siTollip-2: GCGUGGACUCUUUCUAUCU, siTollip-3: GCAUGAUCAACCUGUCAU. Off-target siRNA: UUCUCCGAACGUGUCACGU. Three pairs of siTollip were mixed for use in the experiments.

### Antibodies for western blotting

Endogenous Tollip was detected with a rabbit Tollip polyclonal antibody (Cell Signaling Technology) at a dilution of 1:1000. Myc-tag and flag-tag Tollip were detected with a mouse myc-tag monoclonal antibody (McAb) and a mouse flag-tag McAb (Sigma-Aldrich) at a dilution of 1:5000, respectively. NF-κB p65 was detected with rabbit NF-κB p65 polyclonal antibody (Cell Signaling Technology) at a dilution of 1:2000. α-tubulin and histone H3.1 were detected with a mouse α-tubulin monoclonal antibody (Sigma-Aldrich) at a dilution of 1:10,000 and a rabbit histone H3.1 polyclonal antibody (Abmart Inc, Shanghai, China) at a dilution of 1:5000, respectively. For the preparation of the nuclear and cytoplasmic extracts, cells were collected and separated with NE-PER Nuclear and Cytoplasmic Extracting Reagents (Thermo Scientific) according to the manufacturer’s protocol.

### Flow cytometry to detect HIV-1 reactivation from latency

The HIV-1 latently infected Jurkat T cell C11 clone was kindly donated by Dr. Huan-zhang Zhu (Fudan University, Shanghai, China). The C11 clone harbored an HIV-1 proviral DNA encoding GFP. Upon TNF-α stimulation, the C11 cells expressed GFP as an indication of the reactivation of HIV-1 latency [35]. C11 cells were transfected with either siTollip or the off-target siRNA control for 72 h. The cells were then stimulated with TNF-α (5 ng/ml) for an additional 24 h. The GFP positive populations of C11 cells were detected using a Fortessa flow cytometer (BD Pharmingen) and analyzed with the FlowJo 7.6.1 program.

### Confocal Microscopy

HEK293T cells were seeded on poly-L-lysine-coated slides and transfected with pCMV-Tag3B/Tollip for 24 h. Cells were then fixed with 4% paraformaldehyde (Sigma-Aldrich) for 30 min at room temperature. Then the cells were immunostained with a mouse anti-Myc McAb (Sigma-Aldrich), followed with the secondary antibody of Alexa Fluor 555-labeled goat anti-mouse IgG (Life Technologies). Nuclei were indicated with DAPI. Slides were mounted with Fluorescent Mounting Medium (Dako) and observed using a laser scanning confocal microscope (Leica SP5).

### Statistical analysis

SigmaStat software was used to perform paired *t*-tests.

## Results

### Induction of Tollip expression

**Fig 1:**
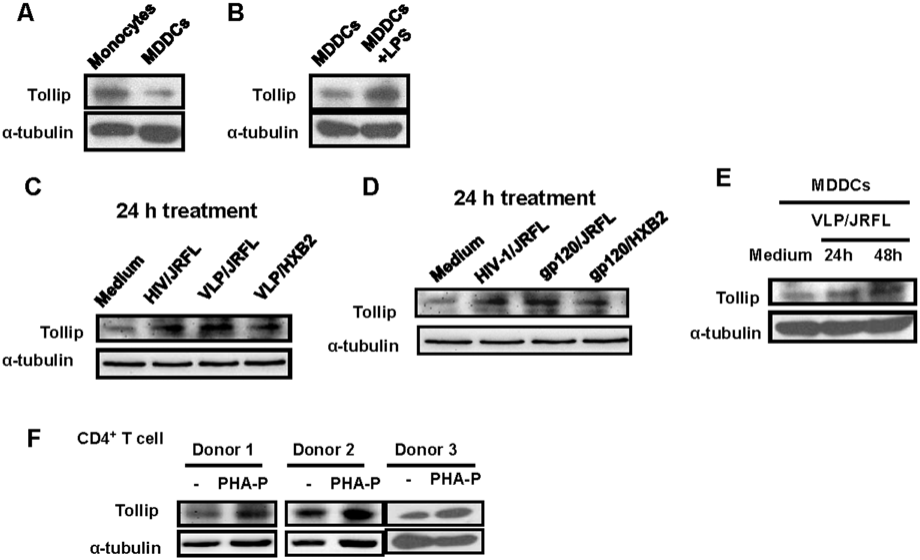
The induced expression of Tollip. (A) Tollip expression in CD14^+^ monocytes and MDDCs, as detected with western blotting using Tollip specific antibodies. (B) LPS treatment induces Tollip expression in MDDCs. 100 ng/ml concentration of LPS was used for 24 h treatment. (C, D, and E) Stimulation with either pseudotyped HIV-1 particles or recombinant gp120 glycoproteins induces Tollip expression in MDDCs. The MDDCs were induced with HIV-luc/JRFL (20 ng p24^gag^), VLP/JRFL (20 ng p24^gag^), or recombinant gp120 glycoprotein (10 μg/ml) derived from either HIV-1/JRFL or HIV-1/HXB2 for the indicated time. Tollip expression was then detected with western blotting using Tollip-specific antibody. (F) The expression of Tollip in resting or PHA-P-activated CD4^+^ T cells.

Tollip showed expression in the CD14^+^ monocytes and MDDCs, and the monocytes displayed a higher expression level (Fig 1A). The stimulation of the MDDCs with LPS (100 ng/ml) for 24 h greatly increased the Tollip expression (Fig 1B). Intriguingly, Tollip was able to be induced for expression by the pseudotyped HIV-1 particles and recombinant gp120 glycoprotein in the MDDCs (Fig 1C–1E). The resting CD4^+^ T cells also expressed high levels of Tollip and could be further stimulated for enhancement by PHA-P treatment for 72 h (Fig 1F).

### Tollip negatively regulates NF-κB activation

**Fig 2:**
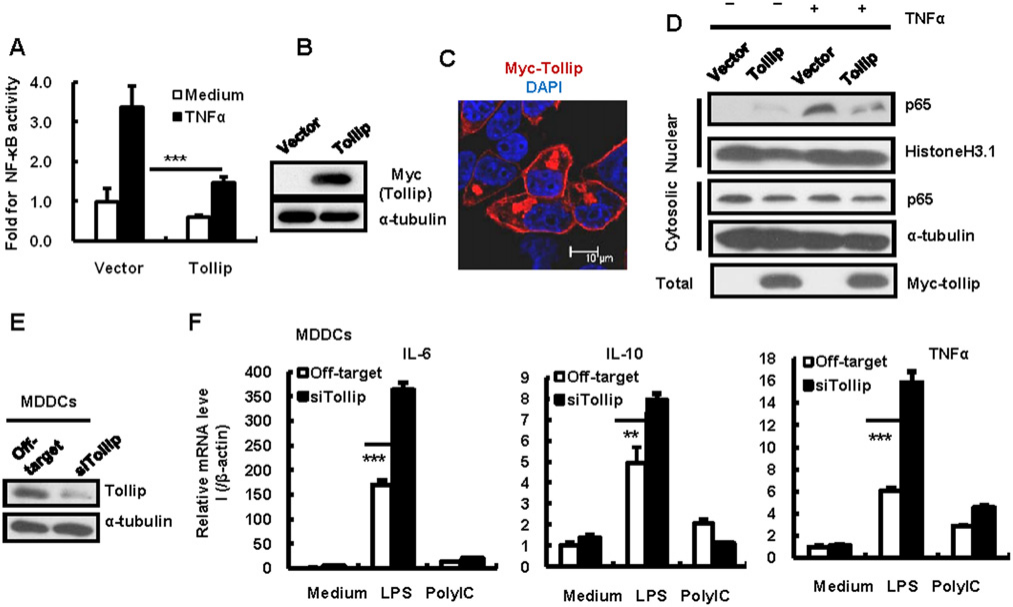
Tollip inhibits NF-κB activation. (A, B) Tollip overexpression inhibits TNF-α-stimulated NF-κB activation. HEK293T cells were co-transfected with pCMV-Tag3B/Tollip (or an empty vector) and p3κB-luc for 24 h. The cells were then stimulated with or without TNF-α (20 ng/ml) for an additional 24 h. Cells were harvested and lysed to detect the 3κB-driven luciferase activity and the Tollip expression. The relative fold for NF-κB activity was calculated based on the luciferase activity. One representative result from five repeats is shown (A). Tollip expression was detected with western blotting for Myc-tag (B). (C) Tollip expression was observed under confocal microscopy. Tollip was indicated in red via immunostained with anti-Myc antibodies, and nucleus was labeled with DAPI and indicated as blue. The merge color was displayed. 10 μm bar is noted. (D) Tollip overexpression blocks NF-κB p65 nuclear import. HEK293T cells were transfected with pCMV-Tag3B/Tollip or an empty vector for 24 h and the cells were stimulated with or without TNF-α (20 ng/ml) for 30 min. The cytoplasmic and nuclear protein extracts were then isolated and NF-κB p65 distribution was detected with western blotting. The proteins of α-tubulin and histone H3.1 were used to denote the cytoplasmic and nuclear extracts, respectively. (E, F) Knocking-down of Tollip in MDDCs promotes LPS-stimulated cytokine expression. The expression of Tollip in the MDDCs was knocked-down with specific siRNAs and detected with western blotting (E). The cells were then stimulated with either LPS (100 ng/ml) or polyI:C (1 μg/ml) for 8 h and the whole cell RNAs were isolated for quantifying the mRNA levels of TNF-α, IL-6, and IL-10. β-Actin was used for the normalization of the mRNA expression (F). **p < 0.01 and ***p < 0.001 are considered significant in a paired *t*-test.

Tollip has been previously reported to negatively regulate TLR-mediated innate immunity[21]. We found that Tollip inhibited NF-κB activation. Transfection of the Tollip-expressing plasmid of pCMV-Tag3B/Tollip in HEK293T cells significantly impaired the TNF-α stimulated NF-κB transcription activation (Fig 2A and 2B). The overexperssed Tollip was mainly distributed in the cytosol, as observed under confoal microscopy (Fig 2C). The role of Tollip in the inhibition of NF-κB activation was further confirmed by detecting the nuclear import of the NF-κB p65 protein using immunoblotting. The nuclear import of the p65 protein induced by TNF-α was blocked in Tollip-overexpressing HEK293T cells (Fig 2D).

We observed the endogenous expression of Tollip in MDDCs, as detected by immunoblotting (Fig 2E). Tollip negatively regulates the IL-1R-, TLR2-, and TLR4-mediated immune responses [20,21]. To verify the negative modulation role of Tollip, the endogenously expressed Tollip was successfully knocked-down in the MDDCs (Fig 2E). The knocking-down of Tollip upregulated LPS-stimulated-TLR4-, but not PolyI: C-stimulated TLR3-mediated cytokine expression (Fig 2F). Taken together, these results confirm that Tollip is a negative regulator of immune response and inhibits NF-κB activation.

### Tollip inhibits HIV-1 LTR-driven transcription

**Fig 3:**
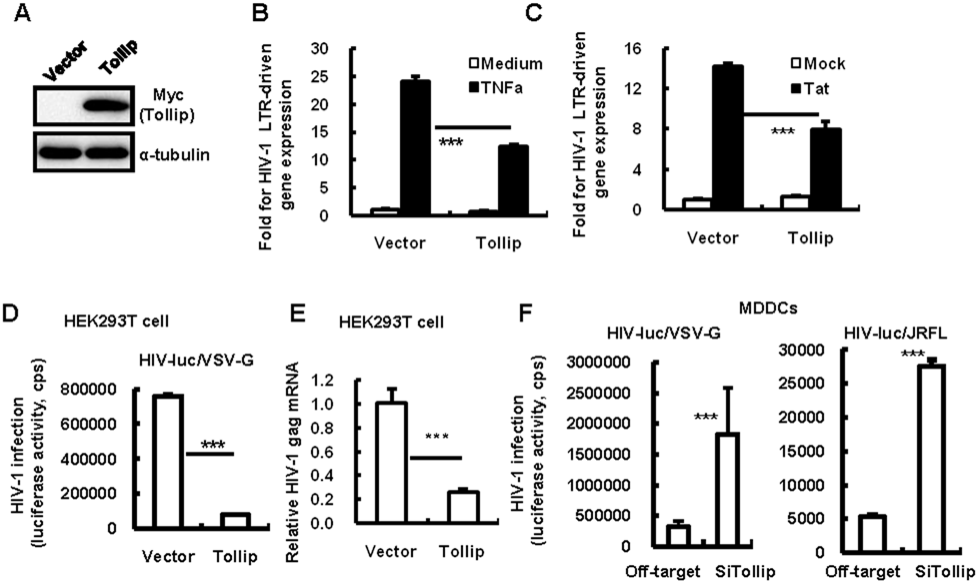
Tollip suppresses HIV-1 infection by inhibiting LTR-driven transcription. (A, B and C) Tollip expression inhibits LTR-driven gene expression. HEK293T cells were co-transfected with pCMV-Tag3B/Tollip (or an empty vector) and the pGL3-luc reporter plasmid containing the full length sequence of HIV-1/NL4-3-LTR, and (C) HIV-1 Tat-expressing plasmid was co-transfected. 24 h post-transfection, cells were treated with TNF-α (20 ng/ml) (B) for an additional 24 h. Tollip expression was detected with western blotting for Myc-tag (A). HIV-1 LTR-driven luciferase activity was measured and the relative enhancement fold was calculated. One representative result from five repeats is shown (B, C). (D, E) Tollip expression inhibits HIV-1 infection. HEK293T cells were co-transfected with pCMV-Tag3B/Tollip or an empty vector. 24 h post-transfection, the cells were infected with HIV-luc/VSV-G (2ng p24^gag^) for an additional 24 h, and viral infection was measured by detecting luciferase activity (D) or by quantifying viral Gag mRNA. One representative result from four repeats is shown (E). (F) Knocking-down of Tollip in MDDCs enhances HIV-1 infection. Tollip-specific siRNAs were transfected in MDDCs for 72 h. The cells were infected with HIV-luc/VSV-G (2 ng p24^gag^) or HIV-luc/JRFL (10 ng p24^gag^). Five days post-infection, viral infection was analyzed by detecting luciferase activity from cell lysates. One representative result from three repeats is shown. ***p < 0.001 is considered significant in a paired *t*-test. Cps, counts per second.

The HIV-1–LTR region usually contains two adjacent NF-κB binding sites that play a crucial role in initiating viral transcription[36–38]. To examine whether the NF-κB inhibition mediated by Tollip confers to the blocking of HIV-1 LTR-promoted transcription, pGL3-luc reporter plasmid containing the full length sequence of HIV-1/NL4-3-LTR was co-transfected with pCMV-Tag 3B-myc/Tollip into HEK293T cells (Fig 3A). The overexpression of Tollip significantly impaired both TNF-α- and HIV-1-Tat-protein-induced HIV-1 LTR-driven gene expression (Fig 3B and 3C). Further, the transfection of Tollip inhibited the infection of single-cycle infectious HIV-1, which was pseudotyped with VSV-G (Fig 3D and 3E). Viral infection was monitored by measuring the luciferase activity or quantifying the production of HIV-1 Gag mRNA (Fig 3D and 3E). Intriguingly, the knocking-down of endogenous Tollip in the MDDCs promoted 5–6 fold greater replication of either HIV-luc/VSV-G or HIV-luc/JRFL (Fig 3F). Taken together, these data demonstrate that Tollip inhibits HIV-1 LTR-driven transcription.

### Tollip mutant that loses the suppression of NF-κB activation abolishes the inhibition of HIV-1 LTR-driven gene expression

**Fig 4:**
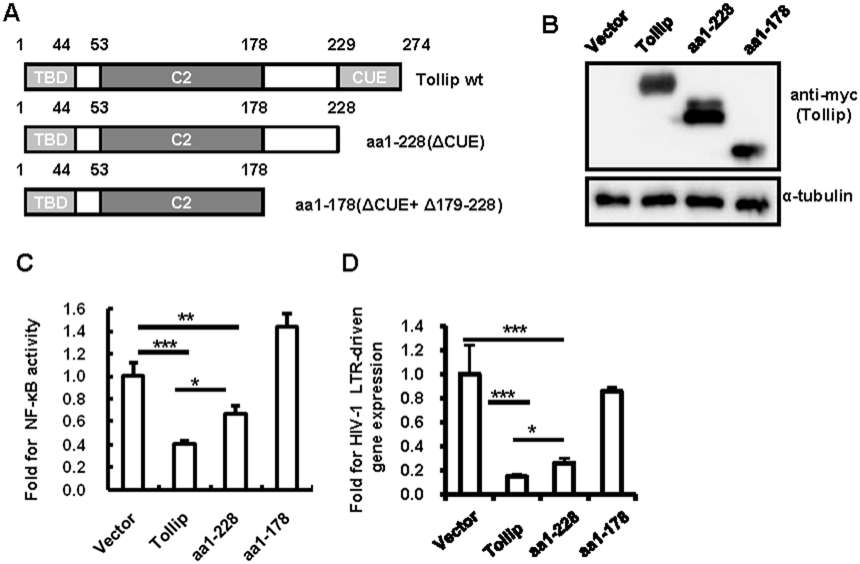
Mapping of the Tollip region required for the inhibition of NF-κB and HIV-1 LTR. (A) The structure diagram of wild type and truncated Tollip protein. Wild type Tollip contains TBD, C2, and CUE domains, and the C-terminal truncations were constructed. (B) The expression of wild type and mutated Tollip in HEK293T cells, as detected with western blotting for Myc-tag. (C, D) The assays of wild type or mutated Tollip for the inhibition on NF-κB activity (C) and HIV-1 LTR-driven transcription (D). One representative result from five repeats is shown. *p < 0.05 and ***p < 0.001 are considered significant in a paired *t*-test.

Tollip contains an N-terminal target of the Myb1 (Tom1) binding domain (TBD), a central conserved 2 (C2) domain, and a C-terminal coupling of ubiquitin to endoplasmic reticulum degradation (CUE) domain[39], as shown in Fig 4A. Two Tollip mutants with varied C-terminal deletion were constructed (Fig 4A and 4B), and the inhibitory effects of these mutants on both NF-κB activation and HIV-1 LTR-driven transcription were compared with that of wild type Tollip in transfected HEK293T cells. As demonstrated previously [19], the deletion of the CUE domain (the mutant ΔCUE or aa1-228) diminished the suppression of NF-κB activation and the further deletion of aa179-228 (the mutant aa1-178) abolished the inhibitory effect (Fig 4C). Correspondingly, the ΔCUE mutant weakened and the aa1-178 mutant abolished the inhibition of TNF-α-stimulated HIV-1 LTR-driven gene expression (Fig 4D). Taken together, these data prove that Tollip-mediated inhibition of HIV-1 LTR-driven transcription is associated with the suppression of NF-κB activation. The linker region between C2 and the CUE domain, which contains the amino acid residues of 179–228, appears to be essential for the inhibition of both NF-κB activation and HIV-1 LTR-driven transcription.

### Tollip silences the post-integrational transcription of HIV-1 proviral DNA and maintains viral latency

**Fig 5:**
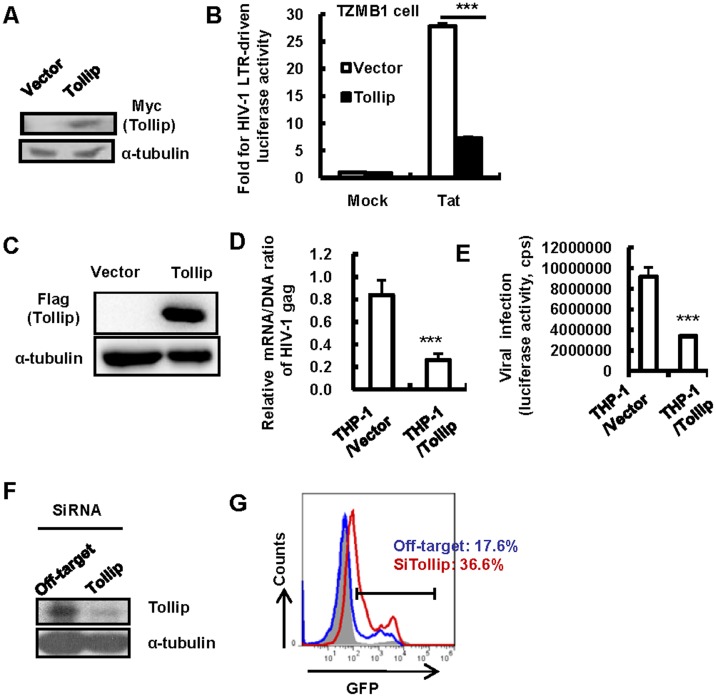
Tollip suppresses the post-integrational transcriptional of HIV-1 proviral DNA and maintains viral latency. (A, B) Tollip suppresses HIV-1 LTR-driven reporter gene expression. TZM-bl cells were co-transfected with pCMV-Tag3B/Tollip (or an empty vector) and pCMV-Tat plasmid for 48 h. The Tollip expression was detected with western blotting for Myc-tag (A), and HIV-1 LTR-driven luciferase activity was measured and the relative enhancement fold was calculated. One representative result from three repeats is shown (B). (C, D and E) Tollip expression inhibits the transcription of HIV-1 proviral DNA. A stable Tollip-expressing THP-1 cell clone was constructed (C) and THP-1/Tollip and THP-1/Vector cells were infected with HIV-luc/VSV-G (20 ng p24^gag^) for 24 h. The total cellular RNA and DNA was then isolated, respectively, for the quantification of HIV-1 Gag mRNA and integrated Gag DNA. The respective nucleic acid amounts were normalized with β-Actin and the ratio of mRNA/DNA of HIV-1 Gag was calculated (D). The level of viral infection was also measured by detecting luciferase activity (E). ***p < 0.001 was considered significant in a paired *t*-test. (F, G) Knocking-down of Tollip increases TNF-α-stimulated HIV-1 reactivation from latency. The HIV-1 latently infected Jurkat cell C11 clone was transfected with Tollip siRNAs or off-target siRNAs for 72 h, then stimulated with TNF-α (5 ng/ml) for an additional 24 h. The Tollip knocking-down was confirmed with western blotting using Tollip-specific antibodies. (F) The reactivation of HIV-1 latency upon TNF-α stimulation was detected with flow cytometry by analyzing GFP expression. The percentage for GFP positive cells is noted (G) and one representative result from three repeats is shown.

The integration of viral DNA into host DNA to form proviral DNA is an essential step in the replication cycle of HIV-1 and other retroviruses. After proving that Tollip inhibits HIV-1 LTR-driven transcription, we next investigated whether Tollip could suppress the post-integrational transcription of HIV-1 proviral DNA. HeLa cell-derived TZM-bl indicator cells, which contain integrated luciferase and ß-galactosidase genes under the control of the HIV-1 LTR promoter, were used for Tollip transfection (Fig 5A). Tollip overexpression in TZM-bl cells diminished Tat-activated, LTR-driven luciferase reporter gene expression (Fig 5B).

To confirm that Tollip inhibited the transcription of the viral proviral DNA, human monocytic cell line THP-1 was infected with pseudotyped HIV-luc/VSV-G. The integrated Gag DNA and its transcribed mRNA were quantified. The stable expression of Tollip in THP-1 cells significantly decreased the production ratio of mRNA versus DNA of HIV-1 Gag (Fig 5C and 5D), confirming the role of Tollip in suppressing the post-integrational transcription of HIV-1. Correspondingly, decreased viral infection was observed, as detected by the virus-harbored reporter gene expression (Fig 5E).

The silencing of HIV-1 LTR-promoted transcription is an important mechanism for maintaining HIV-1 latency [40,41]. CD4^+^ T cells and monocytes are the major reservoirs for harboring latently infected HIV-1[42–47]. Our finding that Tollip mediated the inhibition of HIV-1 LTR-driven gene expression suggested the potential role of Tollip in modulating HIV-1 latency. Thus, we investigated the effect of Tollip on the expression of an integrated HIV-1 proviral DNA in CD4^+^ T-cells. The HIV-1 latently infected Jurkat T-cell clone harbored an HIV-1 proviral DNA encoding GFP. Upon TNF-α stimulation, the C11 cells expressed GFP as an indication of the reactivation of HIV-1 latency [35]. The knocking-down of the Tollip in C11 cells increased TNF-α-stimulated HIV-1 reactivation from latency (Fig 5F and 5G), indicating the role of Tollip in maintaining HIV-1 latency. Taken together, these data demonstrate that Tollip silences the post-integrational transcription of HIV-1 proviral DNA, thereby indicating the role of Tollip in maintaining viral latency.

## Discussion

In this study, we demonstrate that host factor Tollip inhibits HIV LTR-driven gene expression by suppressing NF-κB activation. The silencing of Tollip in the post-integrational transcription of HIV-1 proviral DNA also suggests that Tollip plays a role in maintaining viral latency. Our results reveal the novel role of host factor Tollip in modulating HIV-1 infection and may suggest the hijacking of Tollip as the negative regulator of the TLR pathway, and even the downstream signaling, by HIV-1 in maintaining persistent infection.

Tollip can induce the expression of LPS in MDDCs. The induced expression of Tollip may reflect negative feedback to the TLR4/LPS signaling. In this study, we prove that Tollip inhibits HIV-1 infection, hence the Tollip enhancement in the MDDC/LPS may explain, at least in part, the restriction of HIV-1 replication in LPS-matured MDDCs [48]. HIV-1 recombinant gp120 glycoprotein can also induce Tollip expression in MDDCs. The detailed molecular mechanisms need to be further clarified.

The adaptor protein Tollip was reported to inhibit IL-1R-, TLR2-, and TLR4-mediated signaling[19–21]. Tollip suppresses the activity of IL-1 receptor-associated kinase (IRAK) after TLR activation by inhibiting TLR-mediated signaling [21]. The direct association of Tollip with TLR2 and TLR4 also blocks TLR signaling and the interaction domain of Tollip with TLR4 lies in the C-terminal region between amino acid residues 179–229[21]. Here, we showed that the Tollip C-terminal mutant aa1-178, which was truncated from the residues 179 to terminus, abolished the inhibition on TNF-α-stimulated NF-κB activation. Our data demonstrate the importance of the intact C-terminal region for the Tollip inhibitory role. The CUE domain in the Tollip C-terminal region can be phosphorylated by IRAK-1 kinase[21], but the deletion of the CUE domain did not dramatically reduce the Tollip inhibitory effects on both NF-κB activation and HIV-1 LTR-driven gene expression, suggesting the CUE domain is dispensable for the Tollip inhibitory roles.

We demonstrated the inhibition of Tollip on TNF-α-stimulated NF-κB activation by detecting the 3× NF-κB-driven reporter gene expression and the nuclear import of the NF-κB p65 protein using immunoblotting. Tollip was previously reported to slightly suppress TNF-α-stimulated NF-κB activation in the NF-κB-driven reporter gene expression system[19]. The inconsistency may be due to the different experimental situations and detection methods that were used in the respective studies.

The modulation of TLR signaling on HIV-1 replication remains controversial. It was reported that commensal bacteria such as *Escherichia coli*, *Veillonella parvula*, and *Neisseria mucosa* suppressed HIV-1 expression by stimulating TLR4 signaling, whereas other commensal bacteria such as *Lactobacillus acidophilus*, *Prevotella melaninogenica*, *P*. *bivia*, and *Mycobacterium smegmatis* enhanced HIV-1 infection by stimulating TLR2 signaling[5]. Prothymosin-alpha, a small acidic protein produced and released by CD8^+^ T cells, acts as a ligand for TLR4 and stimulates type I interferon production to potently mediate the post-entry suppression of HIV-1[49]. In contrast, TLR4/LPS signaling activates NF-κB activation for promoting HIV-1 LTR-driven transcription[50]. Tollip is a negative regulator of TLR4 and TLR2 signaling, and whether the negative modulation of Tollip on TLR signaling confers HIV-1 inhibition needs to be further clarified.

Tollip displays high level of expression in monocytes and CD4^+^ T cells. These cells are major reservoirs that harbor latently infected HIV-1 [42–46, 51]. Tollip inhibits HIV-1 LTR-driven transcription by suppressing NF-κB activation. The silencing of HIV LTR-promoted transcription is an important mechanism for maintaining HIV-1 latency[40,41], which reminds us of the need to investigate the potential role of Tollip in maintaining HIV-1 latency. Indeed, the overexpression of Tollip in monocytic cell line THP-1 blocked the post-integrational transcription of HIV-1 proviral DNA, and the knocking-down of Tollip increased the TNF-α-stimulated HIV-1 reactivation in viral latently infected Jurkat CD4^+^ T cells. Given the limitation of HIV-1 latently infected cell line models[51], further use of HIV-1 latently infected primary CD4^+^ T or monocytic cells is required to confirm the role of Tollip in maintaining HIV-1 latency.
